# 

**Published:** 1892-09-24

**Authors:** 


					.PRICE TWOPENCE.
{?813, Vol. XII.?Sept. 24th, 1892.
Wittered at the General Post Office ai a Newspaper (or
tr?wrmi??inn in the United Kingdom and Abroad,]
tH
/ITH EXTRA NURSING SUPPLEMENT
Anmnm, 8s. 6d., post free.
Monthly Parts. 6d.; post free, 8d.
Itto per Annum, 8s., post free.
No. 313. Vol. XII.] Saturday,
September 24th, 1892.
"Twopence.

				

